# Proteinase 3 Interferes With C1q-Mediated Clearance of Apoptotic Cells

**DOI:** 10.3389/fimmu.2018.00818

**Published:** 2018-04-25

**Authors:** Pascale Tacnet-Delorme, Julie Gabillet, Simon Chatfield, Nathalie Thieblemont, Philippe Frachet, Véronique Witko-Sarsat

**Affiliations:** ^1^Université Grenoble Alpes, CEA, CNRS, IBS, Grenoble, France; ^2^INSERM U1016, Cochin Institute, Paris, France; ^3^CNRS UMR 8104, Paris, France; ^4^Université Paris-Descartes, Sorbonne Paris Cité, Paris, France; ^5^Center of Excellence, LABEX Inflamex, Paris, France

**Keywords:** proteinase 3, C1q, apoptotic cells, efferocytosis, autoimmunity

## Abstract

Proteinase 3 (PR3) is the autoantigen in granulomatosis with polyangiitis, an autoimmune necrotizing vasculitis associated with anti-neutrophil cytoplasmic antibodies (ANCAs). Moreover, PR3 is a serine protease whose membrane expression can potentiate inflammatory diseases such as ANCA-associated vasculitis and rheumatoid arthritis. During apoptosis, PR3 is co-externalized with phosphatidylserine (PS) and is known to modulate the clearance of apoptotic cells through a calreticulin (CRT)-dependent mechanism. The complement protein C1q is one mediator of efferocytosis, the clearance of altered self-cells, particularly apoptotic cells. Since PR3 and C1q are both involved in the clearance of apoptotic cells and immune response modulation and share certain common ligands (i.e., CRT and PS), we examined their possible interaction. We demonstrated that C1q binding was increased on apoptotic rat basophilic leukemia (RBL) cells that expressed PR3, and we demonstrated the direct interaction between purified C1q and PR3 molecules as shown by surface plasmon resonance. To better understand the functional consequence of this partnership, we tested C1q-dependent phagocytosis of the RBL cell line expressing PR3 and showed that PR3 impaired C1q enhancement of apoptotic cell uptake. These findings shed new light on the respective roles of C1q and PR3 in the elimination of apoptotic cells and suggest a novel potential axis to explore in autoimmune diseases characterized by a defect in apoptotic cell clearance and in the resolution of inflammation.

## Introduction

Proteinase 3 (PR3) is a neutrophil-derived serine protease located together with its homologs, human neutrophil elastase, and cathepsin G, in azurophilic granules as reviewed in Martin and Witko-Sarsat (1). One particular feature of PR3 is its affinity for membranes leading to its surface expression on viable and apoptotic neutrophils (2). Indeed, PR3 possesses a unique membrane insertion domain composed of four basic (R193, R194, K195, and R227) and four hydrophobic (F180, F181, L228, and F229) amino acids that allows it to anchor to the membrane (3, 4). During neutrophil activation and apoptosis, membrane expression of PR3 increases, and soluble PR3 is also released into the extracellular environment during degranulation (4). PR3 is a pro-inflammatory factor whose membrane expression can potentiate chronic inflammatory diseases such as anti-neutrophil cytoplasmic antibodies (ANCAs) systemic vasculitis (AAV) and rheumatoid arthritis (5). PR3 has been characterized as the autoantigen in granulomatosis with polyangiitis (GPA) (6). During apoptosis, PR3 is co-externalized with phosphatidylserine (PS) *via* its association with phospholipid scramblase 1 (4). Furthermore, it has been proposed that PR3 can modulate apoptotic cell clearance (7) through a mechanism linked to the ability of PR3 to associate with calreticulin (CRT), a protein involved in apoptotic cell recognition and an important “eat-me” signal (8).

Apoptotic cells release “find-me” signals that recruit phagocytes that will recognize, engulf, and degrade them (9, 10) promoting a monocyte/macrophage program that promotes inflammation resolution, tissue repair, and wound healing (11). The function of the complement protein C1q, well known for its role in innate immunity, has been reconsidered over the past 15 years with evidence that it is one mediator of efferocytosis, the mechanism of clearance of altered self-cells and of apoptotic cells in particular (12, 13). C1q serves as a physical bridge between the phagocyte and its prey. Numerous C1q-binding molecules on both sides of the phagocytic synapse have been characterized (14). Among these, cell surface CRT and PS have also been characterized as PR3 partners (7). C1q is a hexamer of heterotrimers, which consists of two typical regions and a collagenous-like fragment of C1q (cC1q) from which six globular regions (GR) [globular region of C1q (gC1q)] emerge. gC1q is involved in the specific recognition of apoptotic cells, and cC1q has primarily been described in C1q recognition by phagocyte membranes (15). However, as the C1q collagenous tail (cC1q) is known to interact with several membrane receptors (14), widely distributed on various cell types, C1q can enter into a vast array of interactions by binding of its heads or/and its stalks depending on their accessibility in a particular situation. Of note, C1q deficiency is strongly associated with autoimmune diseases, such as systemic lupus erythematosus (SLE) and glomerulonephritis and may be associated with compromised removal of apoptotic cells (16).

One other major effect of C1q modulation concerns its function in regulating immune cells, independently from efferocytosis. This includes the role of C1q in neutrophil function. It has previously been shown that the C1q–CRT interaction modulates cytokine release by macrophages, and CRT is released from activated neutrophils (17). Thus, it might be hypothesized that C1q–CRT interaction could also interfere in neutrophil-mediated inflammatory processes.

Given the evidence that PR3 and C1q are involved in both immune response modulation and in clearance of apoptotic cells and share common ligands (i.e., CRT and PS) (7, 18, 19), this study was designed to examine their possible interaction. We investigated C1q binding to apoptotic neutrophils and showed the direct interaction between purified C1q and PR3. To better understand the functional consequence of this partnership, we tested the C1q-dependent phagocytosis of rat basophilic leukemia (RBL) cell line expressing PR3.

These findings shed new light on the respective role of C1q and PR3 in the elimination of apoptotic cells. A number of autoimmune diseases are characterized by defects in apoptotic cell clearance, and this novel potential axis may play a role in the appropriate resolution of inflammation.

## Materials and Methods

### Proteins, Antibodies

C1q was purified from human serum, C1q GR, and the collagen-like region (CLF) were prepared and quantified as described previously (20). Rabbit polyclonal antibody directed against human C1q was from IRPAS group (IBS, Grenoble, France). Mouse monoclonal antibody against C1q (A201) was from Quidel (San Diego, CA, USA), and mouse monoclonal anti-PR3 (clone CLB12.8) was from Sanquin (Amsterdam, Holland). Ficolin 3 was obtained from Nicole Thielens’s team (IBS, Grenoble, France). PR3 was from Athens Research and Technology.

### Blood Cell Isolation, Cells Culture, and Apoptosis Induction

This study was carried out and approved in accordance with the recommendations of the INSERM Institutional Review Board and the Cochin Hospital Ethics Committee (Paris, France). Blood from healthy donors was provided by the Etablissement Français du Sang (Paris, France). Human neutrophils were isolated from EDTA-anticoagulated healthy donor blood using density-gradient centrifugation through polymorphoprep (Nycomed) as previously described (5). To induce physiologic apoptosis, neutrophils were resuspended at 2 × 10^6^/ml in RPMI (Gibco) supplemented with 10% fetal calf serum (FCS) and kept for 16 h at 37°C in a humidified 5% CO_2_ atmosphere. RBL and RBL-PR3 used in this study refers to a previously described cell line (21), transfected with pcDNA3 plasmid alone, or pcDNA3/PR3, respectively. To induce the differentiation of THP1 monocyte cells to macrophages, the cells were treated with 10 nM PMA for 72 h as previously described (20). Apoptosis of RBL cells was induced as follows: briefly, cells were grown in sterile dishes overnight to 60–80% confluence and exposed to 1,000 mJ/cm^2^ UV-B irradiation at 312 nm in a fresh DMEM medium. Cells were then incubated for 17 h at 37°C under 5% CO_2_. Measurement of apoptosis was performed by flow cytometry using the FITC-Annexin V Kit (MACS Miltenyi Biotec) according to the manufacturer’s instructions.

### PR3 and C1q Immunolabeling

RBL cells were washed with PBS and then incubated with C1q (80 μg/ml) during 40 min at 4°C. For all experiments on RBL cells, FcγR blockade was performed with the FcγR-blocking solution (Miltenyi Biotec). Then cells were incubated for 30 min with mouse anti-C1q Abs (A201, Quidel) diluted 1:100. PR3 was detected by a monoclonal anti-PR3 Abs (CLB12.8) at 2 µg/ml. Bound antibodies were visualized with Alexa 488-conjugated goat anti-mouse IgG or cyanine-3 (Cy3) rat anti-mouse IgG. Alexa488-labeled C1qGR and Alexa 647-labeled C1q were prepared using the AlexaFluor-488 and AlexaFluor-647 labeling kits (Invitrogen, ThermoFisher Scientific). For experiments on PMN by confocal microscopy, cells were induced to adhere to poly-l-lysine-precoated coverslip cells. Fixed PMN were incubated with FcγR-blocking solution and then with anti-PR3 Abs, then incubated with Cy3-conjugated anti-mouse IgG before labeling with Alexa488-C1qGR or with Alexa 488-conjugated rat anti-mouse IgG before labeling with Alexa647-C1q (80 μg/ml). Cell slides were mounted glass slides using Vectashield solution with 4′,6′-diamidino-2-phenylindole (Vector Laboratories) and were visualized under a laser spinning-disk confocal microscope (Olympus and Andor, M4D cell imaging platform, IBS). Data were evaluated with Volocity software.

### Uptake of Apoptotic Cells

Rat basophilic leukemia cells were labeled with CFSE (CellTraceTM CFSE Cell Proliferation Kit, Life Technologies) as follows: cells were washed twice and then resuspended at 1 × 10^6^ cells/ml in PBS and incubated with 1 µM CFSE at 37°C for 20 min. The remaining CFSE was quenched with the addition of DMEM-10% FCS for at least 10 min. Cells were then pelleted by centrifugation and resuspended in DMEM-10% FCS before the induction of apoptosis. THP1 cells were labeled with PKH26 dye (Sigma-Aldrich) before PMA induction. Apoptotic RBL cells were then added to THP1-derived macrophages that had been preincubated or not with C1q, at a ratio of 5:1 (RBL:THP1) for 1 h at 37°C, 5% CO_2_ in RPMI medium supplemented or not with 10% of decomplemented FCS. After incubation, cells were washed and harvested with 0.25% trypsin/EDTA and analyzed by flow cytometry (MACSQuant VYB Cytometer, Miltenyi Biotec), and collected data were treated with MACSQuantify software. Phagocytosis was calculated as the percentage of the double CFSE and PKH26 labeled cells in the THP1 macrophage population. Phagocytosis negative controls performed at 4°C or at 37°C in the presence of 5 µM of cytochalasin were subtracted. Significance was tested using non-parametric Wilcoxon signed-rank test for paired samples.

### Surface Plasmon Resonance (SPR) Spectroscopy

Analyses were carried out on a BIAcore 3000 instrument (BIAcore, GE Healthcare). 100 µg of purified PR3 was resuspended in H_2_O to give a final concentration of 1 mg/ml. The running buffer for PR3 immobilization was 10 mM HEPES, 145 mM NaCl, 5 mM EDTA, pH 7.4. PR3 was diluted in acetate buffer (pH 4.5) to achieve a final concentration of 50 µg/ml and was immobilized onto a CM5 sensor chip (GE Healthcare) using the BIAcore amine coupling kit. Binding of C1q, GR, or CLF to immobilized PR3 was measured at a flow rate of 20 ml/min in the running buffer of 50 mM Tris, 150 mM NaCl, 2 mM Ca^2+^ containing 0.005% surfactant P20 (pH 7.4). Surfaces were regenerated by one injection of 5 µl of 20 mM NaOH. The specific binding signal shown was obtained by subtracting the background signal, obtained by injection of the sample over an activated-deactivated surface. Data were analyzed by global fitting to a 1:1 Langmuir binding model of the association and dissociation phases for several concentrations of PR3, using the BIAevaluation 3.2 software (GE Healthcare) and were obtained with a statistic χ^2^ value < 2. The apparent equilibrium dissociation constants (*K*_D_) were calculated from the ratio of the dissociation and association rate constants (*k*_off_/*k*_on_).

### PR3 Proteolytic Activity

Proteinase 3 activity on fibronectin (FN) and C1q was analysis by the method described by Rao et al. (22). Briefly, digestion of purified FN and C1q by PR3 was performed in 0.15 M NaCl, 0.002 M CaCl_2_, 0.01 M HEPES buffer, pH 7.4 at 37°C for 18 h, with an enzyme/substrate molecular ratio of 1:25. The samples were analyzed by SDS-PAGE under reducing conditions. The gel was stained with Coomassie Blue R-250.

## Results

### C1q Binding to Apoptotic Cells Increases With Surface PR3 Exposure

**Figure 1:**
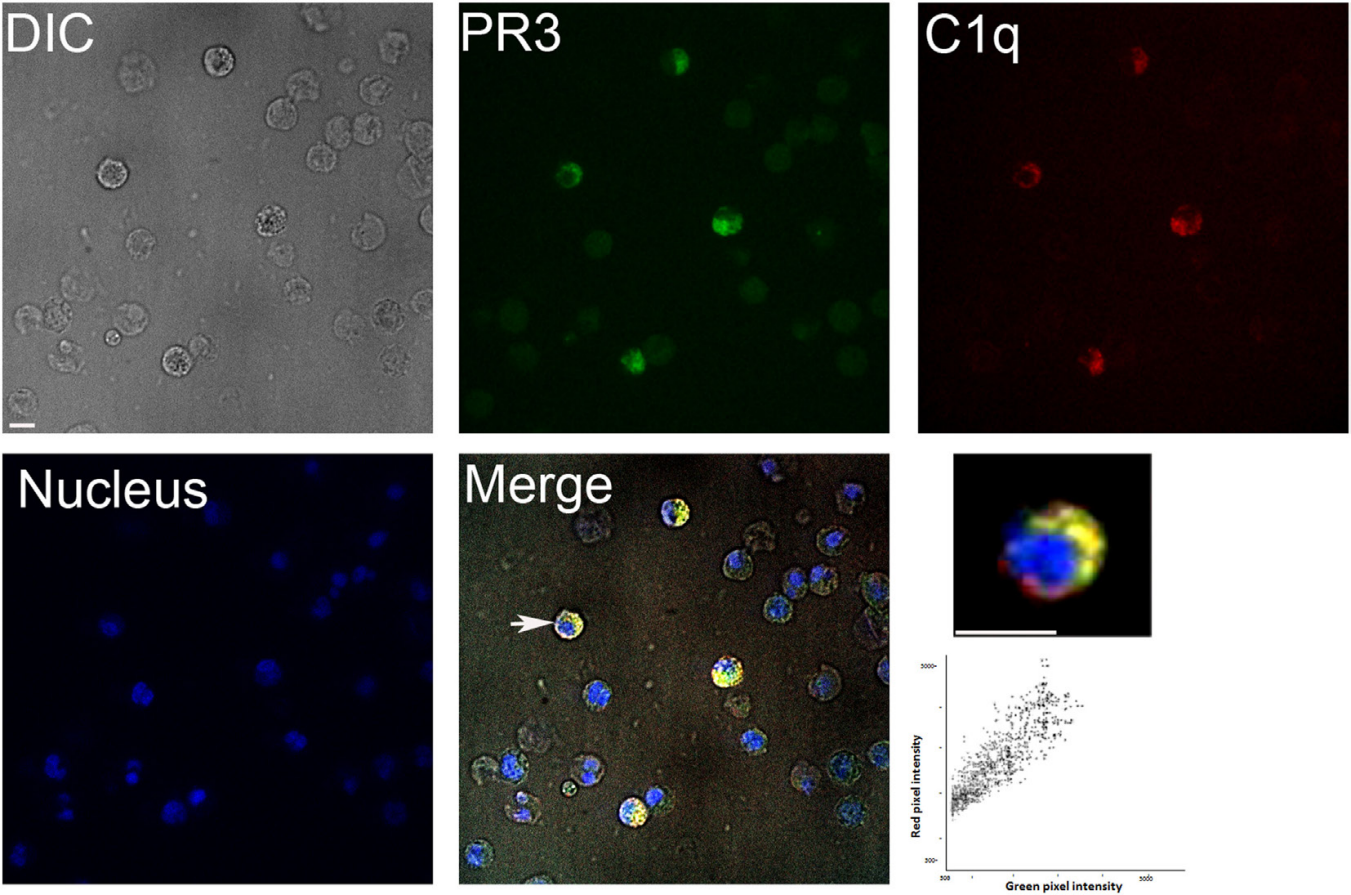
Colocalization of proteinase 3 (PR3) and C1q in neutrophils. Apoptotic neutrophils (prepared as described in Section “Materials and Methods”) were double labeled for membrane PR3 expression and C1q binding using an anti-PR3 mAb followed by an Alexa488-conjugated anti-mouse IgG and then incubating cells with Alexa647-C1q. Nuclei were labeled with DAPI. Samples were visualized by confocal microscopy under differential interference contrast (DIC), DAPI, A488, and A647 filters, and merge is shown (as indicated). Higher magnification is shown for one selected cell indicated by a white arrow. Scatterplot of red and green pixel intensities collected from the focal plane of the cell shown is represented. Scale bar 8 µm. Colocalization (yellow regions in merge) was evaluated by Pearson’s correlation coefficient ≥0.9.

**Figure 2:**
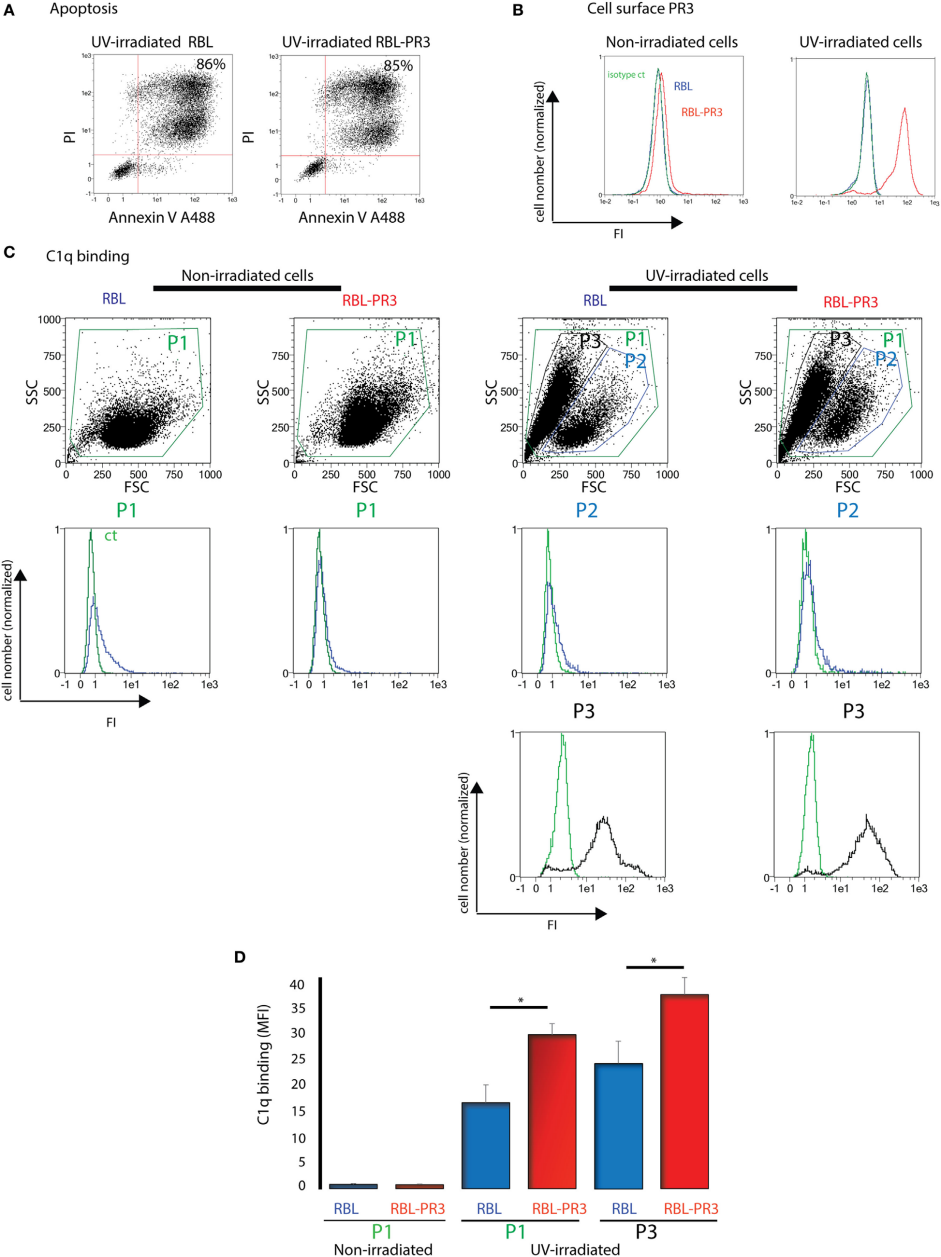
C1q binding increases on apoptotic cells that express proteinase 3 (PR3) on their surface. **(A)** Apoptosis of rat basophilic leukemia (RBL) and RBL-PR3 was analysed by double Annexin V/PI labeling. FSC/SSC dot plot of the irradiated population is shown, and gating strategy used for AV/PI analysis is indicated. Percentages correspond to the AV/PI positive population. **(B)** Untreated and UV-B irradiated RBL and RBL-PR3 cells were labeled with anti-PR3 antibody. Isotype controls in green for PR3-RBL cells are shown. **(C)** Cells were incubated with soluble C1q, and its binding was detected using a monoclonal antibody against C1q. Representative experiments are shown. Blue curves correspond to C1q binding on P1 gate (non-irradiated cells) or on P2 gate (UV-irradiated cells). Black curves represent the fluorescence of the P3 gate which appears with apoptosis; green curves represent the fluorescence of cells in the same gate in the absence of C1q. **(D)** Quantification of the C1q binding as shown in panel **(C)**, done on the P1 and P3 subsets (three independent experiments, *P < 0.05, Student’s *t*-test). Analyses were monitored by flow cytometry as described in Section “Materials and Methods.” Abbreviations: FI, fluorescence intensity; MFI, median fluorescence intensity; SSC, side scatter; FSC, forward scatter.

We first examined the colocalization of C1q and PR3 at the surface of apoptotic neutrophils from healthy human donors. As shown in Figure 1, under physiologic apoptosis induction, apoptotic neutrophils were recognized by C1q and PR3 partially colocalized with C1q. Similar colocalization was also observed using the C1q globular head (not shown). As it has been shown that PR3 expression on the neutrophil population is non-homogenous and is characterized by important interindividual variability, further experiments were conducted using the previously established RBL cell line that expresses PR3 at the cell surface during apoptosis (7). Late apoptosis was induced in RBL-PR3 and RBL (transfected with the empty plasmid) cells using UVB irradiation and demonstrated by double Annexin V/PI labeling (Figure 2A). Immunolabeling experiments of RBL cells confirmed that wild-type RBL cells did not express PR3 (Figure 2B, blue curves). PR3 was significantly externalized at the surface of apoptotic RBL-PR3 cells whereas almost no PR3 was detected on untreated PR3-RBL cells (Figure 2B, PR3 labeling, red curves). In addition, we analyzed the C1q binding to these cell populations. As expected from its capacity to recognize apoptotic bodies, C1q bound to both RBL and PR3-RBL cell subsets which appeared after UV irradiation (Figure 2C, P3 subset). However, we observed an increase of the C1q binding to apoptotic PR3-RBL compared with RBL cells (Figures 2C,D). Together, these observations prompted us to investigate the direct PR3–C1q interaction.

### Purified C1q Binds PR3

**Figure 3:**
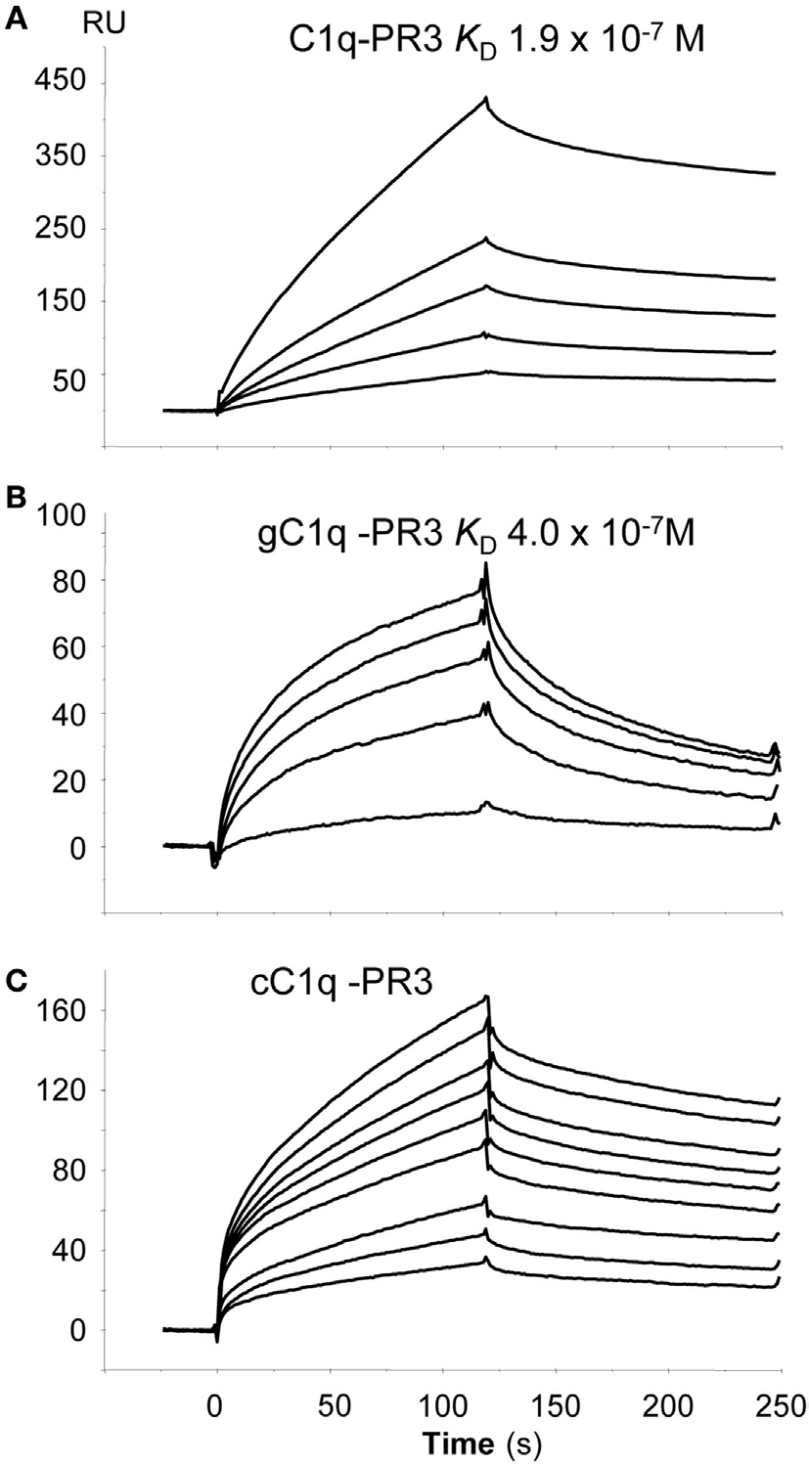
Globular region of C1q (gC1q) and collagenous-like fragment of C1q (cC1q) fragments bind to proteinase 3 (PR3). Binding of intact C1q **(A)**, of the globular C1q heads, gC1q **(B)**, and of the collagen tail of C1q, cC1q **(C)** to immobilized PR3. All interactions were measured in the running buffer at a flow rate of 20 µl/min. Association and dissociation curves were each recorded for 120 s. The concentrations of soluble ligands were as follows: C1q: 0.05, 0.10, 0.20, 0.30, and 0.50 µM; gC1q 0.10, 0.40, 0.70, 0.80, and 1.0 µM; cC1q 0.3, 0.4, 0.5, 0.7, 0.8, 0.9, 1.0, 1.2, and 1.4 µM. The kinetic parameters of the interactions were determined by recording sensorgrams at varying concentrations are listed in Table 1. All other conditions are described in Section “Materials and Methods.”

To further investigate whether there is a direct interaction between PR3 and C1q, SPR experiments were performed using purified PR3 immobilized on a sensor chip. Intact C1q, its globular heads (gC1q), or its collagenous tail (cC1q) were used as soluble ligands. As illustrated in Figure 3, intact C1q, its gC1q and cC1q regions, all bound to immobilized PR3 while no interaction was detected using the C1q-related protein Ficolin 3 (data not shown). The kinetic parameters of PR3–C1q interaction were determined by recording sensorgrams at varying ligand concentrations. For intact C1q and its globular heads, the kinetic (*k*_a_, *k*_d_) and dissociation (*K*_D_) constants were calculated with a simple 1:1 Langmuir binding model (Table 1) with *K*_D_ values of 1.9 × 10^−7^ and 4.0 × 10^−7^ M, respectively. Thus, the *K*_D_ value of the interaction with PR3 was significantly lower for the full-length C1q compared with its isolated GR, accounting for an increased binding avidity of C1q arising from its hexameric structure. In addition, cC1q bound in a dose-dependent manner to PR3. Although the curve fitting was not adequate to determine accurately the kinetic value of this interaction, this result suggests that components of the collagen tail of C1q could also be involved in the interaction.

**Table 1:** Kinetic constants for the binding of C1q to immobilized proteinase 3 (PR3).

Soluble analyte	PR3		
	*k*_on_ (M^−1^ s^−1^)	*k*_off_ (s^−1^)	*K*_D_ (M)
C1q	7.8 × 10^3^	1.51 × 10^−3^	1.9 × 10^−7^
gC1q (globular domain)	1.8 × 10^4^	7.2 × 10^−3^	4.0 × 10^−7^
cC1q (collagenous-like fragment)	nd	nd	nd

*Binding of C1q, its globular region of C1q (gC1q), and collagenous-like fragment of C1q (cC1q) to PR3 were measured as described in Section “Materials and Methods.” The association (*k*_on_) and dissociation (*k*_off_) rate constants were determined by global fitting of the data using a 1:1 Langmuir binding model (A + B ⇔ AB, BIAevaluation 3.2). The dissociation constants *K*_D_ were determined from the *k*_off_/*k*_on_ ratios. The data presented were obtained with a statistic χ^2^ value < 2*.

*ND, not determined (i.e., despite the dose-dependent response the *K*_D_ value cannot be determined satisfactorily)*.

### PR3 Impaired C1q Enhancement of Apoptotic Cell Uptake

**Figure 4:**
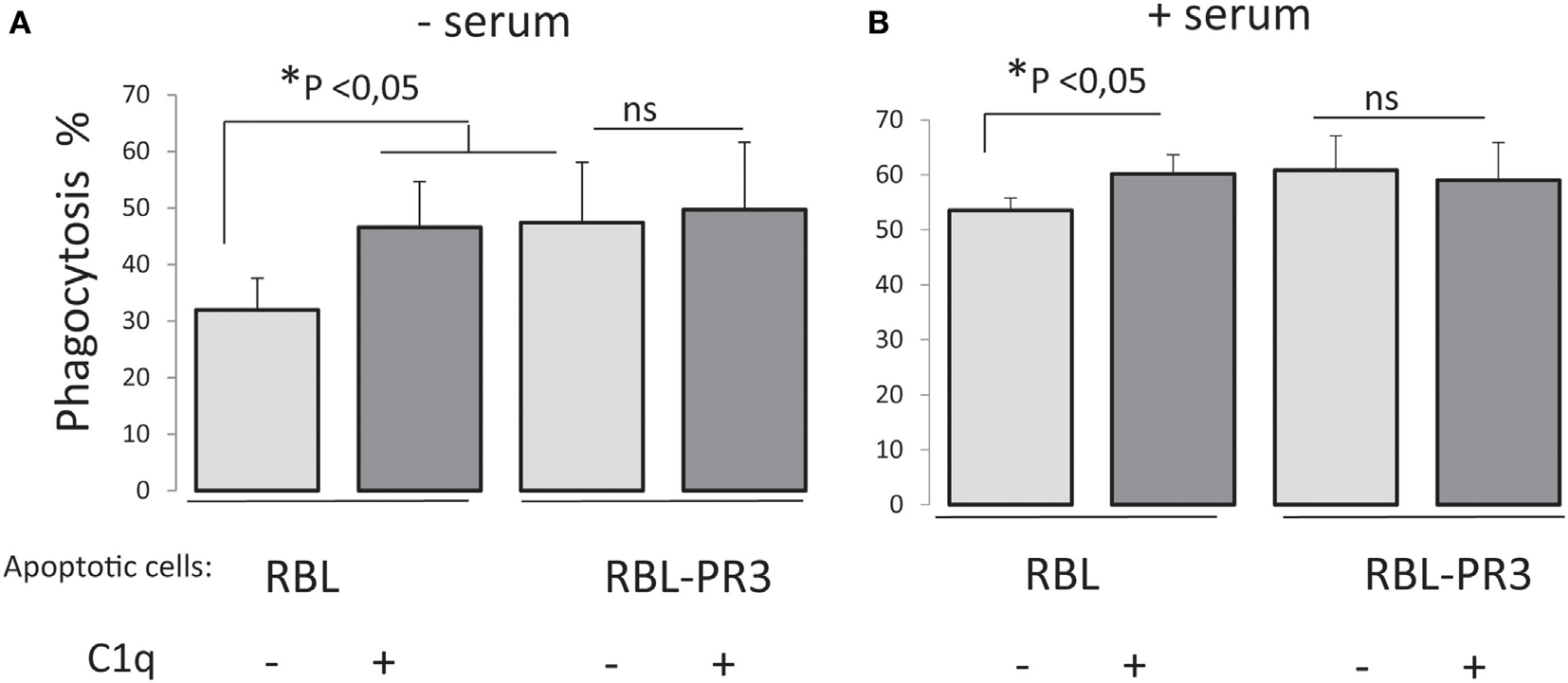
Surface proteinase 3 (PR3) exposure supresses C1q enhancement of apoptotic cell phagocytosis. Late apoptotic rat basophilic leukemia (RBL) cells labeled with CFSE, opsonized or not with C1q (80 µg/ml), were incubated with PMA-treated THP1 cells labeled with PKH26 dye, 1 h at 37°C in absence **(A)** or in presence of serum **(B)**. Phagocytosis is expressed as the percentage of the double-labeled cells in the macrophage population (i.e., PKH26 and CFSE labeled cells). Data are the mean ± SD of independent experiments. *Significance was tested using non-parametric two tail Wilcoxon signed-rank test for paired samples, *n* = 5 (without serum) and *n* = 4 (with serum).

To analyze the possible PR3/C1q-dependent effect on the uptake of apoptotic cells by macrophages, we measured the capacity of PMA-stimulated THP1 cells to phagocytose PR3-expressing RBL cells. Late apoptotic RBL cells or RBL-PR3 cells were added to THP1 macrophages, and their uptake was determined by flow cytometry. To assess the effect of C1q on phagocytosis specifically, whole C1q at a physiological concentration (80 µg/ml) was added to opsonize apoptotic cells before macrophage–target cell contact. RBL cells were efficiently engulfed by THP1 macrophages, and the phagocytosis increased significantly with C1q either in presence or absence of serum (Figure 4). Remarkably, this C1q enhancement of phagocytosis was abolished for PR3-expressing cells as we did not observe any effect of C1q on the uptake of RBL-PR3 cells. In addition, we observed that apoptotic RBL-PR3 cells were more readily phagocytosed by THP1 macrophages than the RBL cells in absence of serum (*P* = 0.043, *n* = 5). Interestingly, this difference is not apparent when experiments were done in presence of serum (*P* = 0.108, *n* = 4). This is possibly due to the presence of serum protein(s) that could interfere with PR3.

### C1q Was Not Proteolytically Cleaved by PR3

**Figure 5:**
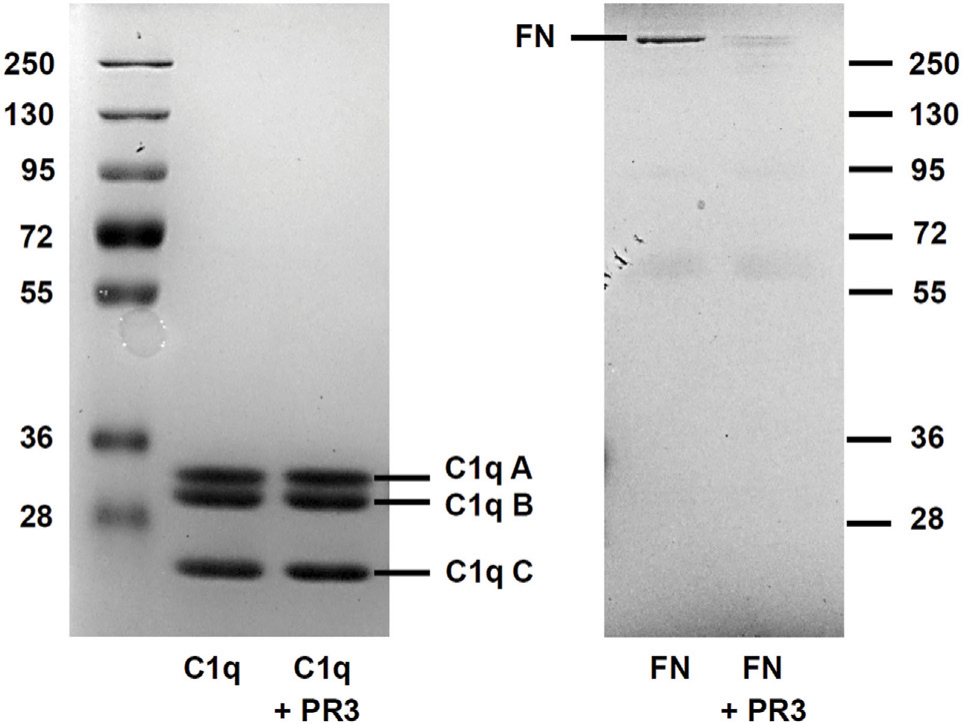
C1q is not cleaved by proteinase 3 (PR3). Fibronectin (FN) and C1q were submitted to PR3 digestion at 37°C for 18 h. The digestion products were separated on a 10% SDS-PAGE gel under reducing conditions and visualized by Coomassie staining. The position of the three (A–C) subunits of C1q and of FN is shown.

As PR3 is able to cleave a broad range of matrix proteins such as elastin, FN, or laminin, we tested the hypothesis that PR3 could degrade C1q and thus impair its ability to enhance phagocytosis. After 18 h of incubation, PR3 did not degrade C1q but efficiently cleaved FN used as a control (Figure 5). This suggests that proteolytic cleavage of C1q was not the mechanism explaining the failure of C1q to enhance uptake of apoptotic RBL-PR3 cells.

## Discussion

This study provides the first experimental evidence that C1q, well known to be involved in the recognition of apoptotic cells and their uptake by phagocytes interacts with the neutrophil-specific serine protease PR3 with potentially relevant consequences for apoptotic cell uptake. Our conclusion is based on the following observations: (1) C1q and PR3 colocalize at the surface of neutrophils; (2) C1q binds more efficiently apoptotic RBL cells when they expose PR3 at their surface and; (3) C1q recognizes PR3 with a sub-micromolar affinity (*K*_D_ = 1.9 × 10^−7^ M) as shown by SPR analysis. The ability of C1q to bind PR3 on the cell surface is further substantiated by the effect of PR3 exposure on the phagocytosis of apoptotic cells in the presence of C1q. Remarkably, when apoptotic RBL cells express cell surface PR3, the C1q effect on their uptake is undetectable in contrast to the wild-type RBL cells and what has previously been observed with other apoptotic cell types (13, 23). The mechanism of inhibition does not appear to be simple cleavage of C1q by the protease activity of PR3. It should be noted that in this phagocytosis model, PR3 expression did not impair the uptake of apoptotic cells even in absence of C1q. However, together with the characterization of the PR3–C1q direct interaction, it might suggest that PR3 could affect C1q-mediated functions.

Interestingly, we have previously observed using an *in vivo* phagocytosis assay of RBL cells in mice, that PR3 membrane expression efficiency decreases the phagocytosis of apoptotic cells by peritoneal macrophages (7) in contrast to the increased phagocytosis *in vitro* using macrophages differentiated from the THP1 monocyte cell line in this study. We hypothesize that PR3 could bind to other mediators implicated in the phagocytosis process, e.g., serum proteins such as C1q. Our present study showing that C1q-mediated enhancement of phagocytosis is impaired when cells expose PR3 on their surface supports this hypothesis and strongly suggests that PR3 binding to C1q disables C1q-mediated phagocytic macrophage function. We have also observed differences between phagocytosis in the presence and absence of serum, indicating that other serum molecules could interfere with this process in the specific tissue environment. Of note, it has been demonstrated that C1q and PR3 share common binding partners involved in efferocytosis, the well-known “eat-me signals” and immune modulators CRT and PS (7, 18, 19, 24, 25). Importantly, we have previously shown that the ectoCRT–C1q interaction modulates the efferocytosis process. As co-externalization of CRT and PR3 occurs during neutrophil apoptosis, this presumably could interfere with the C1q-dependent PR3 effect on phagocytosis. These findings should be taken into consideration when interpreting the role of PR3 under pathophysiological conditions.

Neutrophils play a key role in the molecular pathology of number of autoimmune diseases since these cells can generate neo-epitopes that have the potential to break immune tolerance resulting in the generation of autoantibodies (26). Of particular interest, PR3 is the major target of ANCA in GPA, and a high percentage of neutrophils bearing membrane PR3 is considered a risk factor for autoimmune vasculitis. Indeed, we have previously proposed that PR3 can inhibit the clearance of apoptotic neutrophils by phagocytes, acting as a “don’t eat me” signal by interfering with the ability of CRT to promote phagocytosis (7) and perturbing the normally anti-inflammatory response following phagocytosis of apoptotic cells by macrophages (27). Furthermore, PR3 acts as an alarmin inducing pro-inflammatory cytokine and chemokine production by macrophages and dendritic cell activation leading to dysregulated T cell polarization and favoring autoimmunity (27). Another key protein of immune tolerance is C1q through its facilitation of apoptotic cell clearance. Indeed, C1q deficiency is linked to development of autoimmune diseases including SLE, probably through the alteration of efferocytosis (28). Genetic variants in the region of the C1q genes have also been associated with rheumatoid arthritis (29). Interestingly, the frequency of circulating PR3-high neutrophils also increases in patients with rheumatoid arthritis (5). Our current data reinforce the idea that the PR3 pro-inflammatory effect could be mediated by its ability to specifically bind molecules involved in the safe removal of apoptotic cells. On the other hand, the variability of PR3 expression on the neutrophil population raises the question of its physiological role. It is tempting to speculate that PR3 membrane exposure could be associated with subpopulations of neutrophils that exhibit specific regulatory functions linked to innate clearance or inflammatory response.

**Figure 6:**
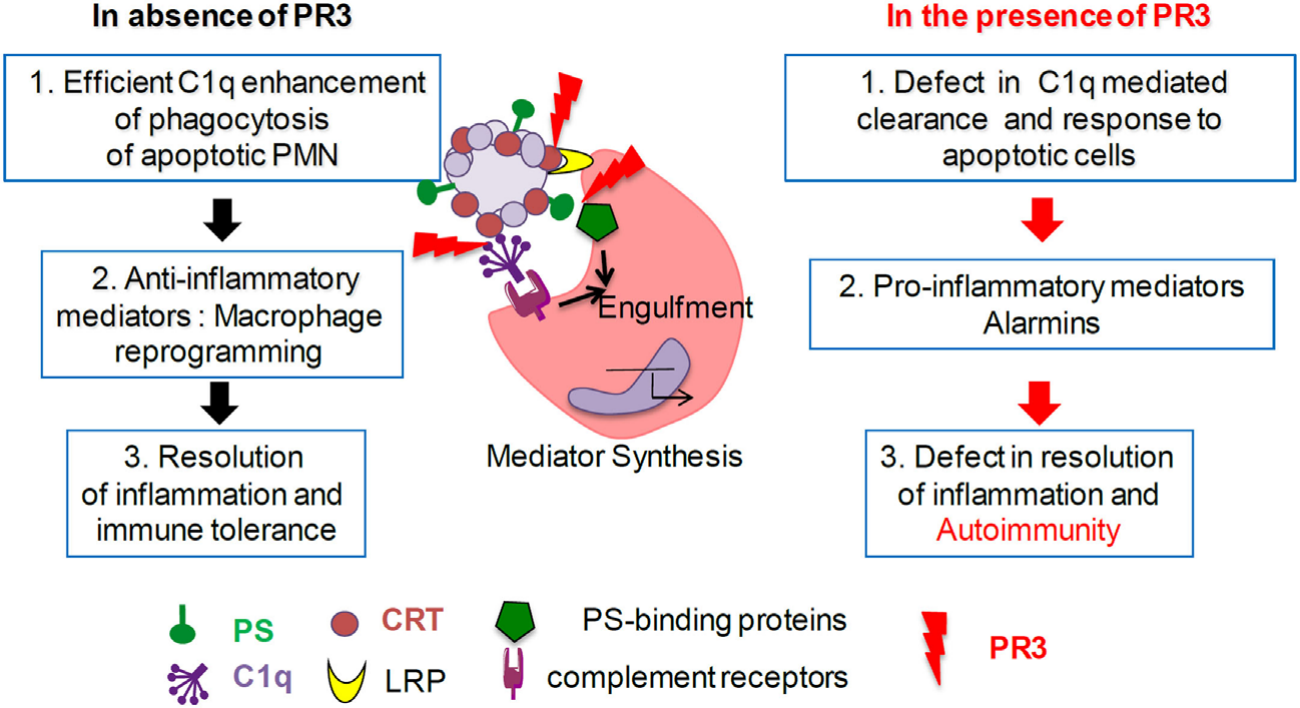
Proteinase 3 (PR3) expression on apoptotic cells decreases C1q-mediated phagocytosis. *In absence of membrane PR3*, C1q opsonization of apoptotic cells through apoptotic cell-associated molecular pattern recognition [such as phosphatidylserine (PS), calreticulin (CRT), and others] enhances phagocytosis through macrophage receptors (such as LRP and others) and triggers anti-inflammatory responses leading to the resolution of inflammation. *In the presence of membrane PR3*, expression on apoptotic cells, PR3 that binds PS, CRT, and C1q, could interfere with the multimolecular complexes at the interface between apoptotic neutrophils and macrophage, delaying or affecting phagocytosis of apoptotic cells, activating a pro-inflammatory immune response favoring autoimmunity.

In summary, together with previously published data, our findings highlight the existence of interactions between key serum and tissue proteins involved in the efferocytosis process (recognition and/or phagocyte signaling pathways) including multivalent proteins (e.g., C1q) with a propensity to aggregate molecules (Figure 6). We hypothesize that regulation of the immune response and the initiation of pathological events may be dependent on the relative abundance of these proteins. Efficient apoptotic body removal has important inflammatory consequences, and these molecules may be important to target in neutrophil-associated autoimmune disorders.

## Ethics Statement

This study was carried out and approved in accordance with the recommendations of the INSERM Institutional Review Board and the Cochin Hospital Ethics Committee (Paris, France). Blood from healthy donors was provided by the Etablissement Français du Sang (Paris, France).

## Author Contributions

PF and VW-S designed the study and analyzed and interpreted data. JG, NT, and PT-D contributed to the study design and acquired, analyzed, and interpreted data. PF, NT, SC, and VW-S wrote the manuscript. All the authors approved the submitted version.

## Conflict of Interest Statement

The authors declare that the research was conducted in the absence of any commercial or financial relationship that could be considered a potential conflict of interest.

## Abbreviations

ANCA, anti-neutrophil cytoplasmic antibody; CRT, calreticulin; cC1q, collagenous-like fragment of C1q; gC1q, globular region of C1q; GPA, granulomatosis with polyangiitis,; PS, phosphatidylserine; PLSCR1, phospholipid scramblase 1; PR3, proteinase 3; RBL, rat basophilic leukemia; SPR, surface plasmon resonance.
